# Survey research with a random digit dial national mobile phone sample in Ghana: Methods and sample quality

**DOI:** 10.1371/journal.pone.0190902

**Published:** 2018-01-19

**Authors:** Kelly L’Engle, Eunice Sefa, Edward Akolgo Adimazoya, Emmanuel Yartey, Rachel Lenzi, Cindy Tarpo, Nii Lante Heward-Mills, Katherine Lew, Yvonne Ampeh

**Affiliations:** 1 School of Nursing and Health Professions, University of San Francisco, San Francisco, California, United States of America; 2 FHI 360, Communicate for Health (C4H) Project, Accra, Ghana; 3 FHI 360, Behavioral, Epidemiological, and Clinical Sciences Division, Durham, North Carolina, United States of America; 4 VOTO Mobile, Accra, Ghana; 5 FHI 360, Strategic Information and Monitoring & Evaluation Division, Washington, D.C., United States of America; 6 Ghana Health Service/Ministry of Health, Health Promotion Department, Accra, Ghana; Massachusetts General Hospital, UNITED STATES

## Abstract

**Introduction:**

Generating a nationally representative sample in low and middle income countries typically requires resource-intensive household level sampling with door-to-door data collection. High mobile phone penetration rates in developing countries provide new opportunities for alternative sampling and data collection methods, but there is limited information about response rates and sample biases in coverage and nonresponse using these methods. We utilized data from an interactive voice response, random-digit dial, national mobile phone survey in Ghana to calculate standardized response rates and assess representativeness of the obtained sample.

**Materials and methods:**

The survey methodology was piloted in two rounds of data collection. The final survey included 18 demographic, media exposure, and health behavior questions. Call outcomes and response rates were calculated according to the American Association of Public Opinion Research guidelines. Sample characteristics, productivity, and costs per interview were calculated. Representativeness was assessed by comparing data to the Ghana Demographic and Health Survey and the National Population and Housing Census.

**Results:**

The survey was fielded during a 27-day period in February-March 2017. There were 9,469 completed interviews and 3,547 partial interviews. Response, cooperation, refusal, and contact rates were 31%, 81%, 7%, and 39% respectively. Twenty-three calls were dialed to produce an eligible contact: nonresponse was substantial due to the automated calling system and dialing of many unassigned or non-working numbers. Younger, urban, better educated, and male respondents were overrepresented in the sample.

**Conclusions:**

The innovative mobile phone data collection methodology yielded a large sample in a relatively short period. Response rates were comparable to other surveys, although substantial coverage bias resulted from fewer women, rural, and older residents completing the mobile phone survey in comparison to household surveys. Random digit dialing of mobile phones offers promise for future data collection in Ghana and may be suitable for other developing countries.

## Introduction

Demographic and health survey (DHS) programs have been relied on to assess population health in low and middle income countries (LMIC) since 1984. Implemented in more than 90 countries, standard DHS are repeated in many countries every 3–6 years to assess a range of monitoring and evaluation indicators of population, health, and nutrition. DHS surveys have large, nationally representative samples that are generated through household interviews.[1] Yet, DHS are time-consuming and expensive.[2] They require door-to-door in-person interviews, suitable transportation for poor roads, and multiple attempts to reach selected households.[3] Interviewer training and supervision is time-intensive and requires ongoing quality assurance efforts.[3,4] Given the many resources needed for robust implementation of DHS, alternative data collection methodologies are warranted.

New vehicles for collecting data are rapidly becoming available in the form of mobile phones. Mobile phone penetration rates are high globally and in LMIC, where 8 in 10 people own a mobile phone.[5] Data from 2014–2015 show that in Sub-Saharan Africa, between 61[6] to 73%[5] of people own mobile phones, and these rates continue to increase. Random digit dialing (RDD) of telephone numbers is commonly used to generate a representative sample for population research. Traditionally used in countries with high landline phone coverage, RDD sampling is usually faster, increases accessibility to respondents, and produces data that is less subject to interviewer effects compared to household interviews.[7] RDD surveys on mobile phones may provide similar benefits to RDD surveys on landlines, especially in developing countries like Ghana where landline telephones are few but wireless mobile phones are numerous.[5,6, 8,9]

However, little data are available on representativeness and sample quality using RDD mobile phone survey methods in LMIC.[10] Most LMIC have sizeable rural populations, large geographic distances between population areas, and rapidly increasing mobile phone coverage—suggesting that RDD mobile phone surveys may provide an advantageous method for population health research. Investigating data collection procedures and sample quality using these methods can inform new approaches to conducting population research as well as survey research with high-interest socio-demographic groups in countries like Ghana where mobile phone penetration rates are high [6].

The Communicate for Health (C4H) Program in Ghana is a 5-year national behavior change communication and health promotion campaign implemented collaboratively by the Health Promotion Department of the Ghana Health Service (GHS), FHI 360, and partners. The campaign uses mass media, social and digital media, and community radio programming to effect improvements in family planning, water, sanitation, and hygiene, nutrition, maternal and child health, malaria prevention and case management, and HIV/AIDS. The campaign is evaluated through a multi-wave, interactive voice response (IVR), RDD mobile phone survey. We use C4H baseline survey data to assess call outcomes and response rates and evaluate sample biases due to errors in coverage and non-response. The Ghana DHS[11] and the Ghana Statistical Survey (GSS) Population and Housing Census [12] are used to benchmark results.

## Materials and methods

### Data collection

The RDD technique used random number generators to construct potential phone numbers using the 12-digit basic structure of mobile phone numbers in Ghana. The first three digits corresponded to the international country calling code for Ghana (233), followed by two digits that indicated assigned prefixes for the mobile network operators (MNOs), and the remaining seven numbers were randomly generated. Calls were placed between 8am and 8pm local time, but heavy volume call times (e.g., weekdays between 10am-12pm and 5-7pm) were avoided based on agreement with MNOs. Respondents who missed the call or were unable or unwilling to complete the survey at the time of the call could call back using their phone’s missed call or redial feature to take the survey at a more convenient time. Each number was dialed only once.

Any person who answered the phone was eligible for survey participation if they were at least 18 years old. Data were collected for 27 days between 17 February and 15 March 2017. A total of 13,016 interviews were completed.

### Ethics approval

The study protocol was approved by the FHI 360 Protection of Human Subjects Committee (IRB000000793) and the Ghana Health Services. All survey participants provided informed consent for study participation.

### Survey design

The first half of the interview covered demographics, followed by radio and TV viewing, exposure to health messages, and use of insecticide treated bednets (ITN). Respondents were asked between 16 and 19 questions total, depending on their pregnancy status and age of children. When possible, questions were worded to match other surveys such as the DHS, although some questions required rephrasing to ensure comprehension in the IVR format.

The interview was pretested with a series of A/B tests to compare the impact of varying language, order, and formatting on survey response. First, tests of survey language showed that using the most widely spoken local language in Ghana—Twi—for the brief greeting message that also identified GHS as the sponsor, and then providing choice of survey language in a random instead of fixed order, yielded a higher call continuation rate and more diversity in respondent region. Second, a shorter, straightforward message introducing the survey and providing essential elements of informed consent yielded higher call continuation than longer introduction messages that included an emotional appeal for participation. Third, shorter questions were less likely to be repeated than longer items that included response options in the question wording, and multiple choice format (e.g., age categories) improved call continuation and data quality in comparison to entering a specific number (e.g., 24).

The IVR survey involved listening to a pre-recorded question asked in a local language selected by the respondent, and then answering through keypad presses on the mobile phone. A question could be repeated by pressing ‘0.’ Answering the call was free to respondents, and free callbacks could be made to complete the interview at the respondent’s convenience. Before beginning, respondents were told the call was free, names would not be collected, data were confidential, and participants must be 18 or older. Informed consent was indicated by asking respondents to press ‘1’ to continue with the call. The survey script in English is presented in S1 Appendix.

### Call outcomes and response rates

Response rates were computed according to the American Association of Public Opinion Research (AAPOR) guidelines.[13] The final dispositions of the mobile phone telephone numbers were classified using AAPOR standard definitions (Table 1). We computed ‘e’ as the proportion of all callers screened for eligibility who were eligible. Completed interviews were defined as answering all questions asked. Partial interviews were defined as answering the first half of the survey that included demographic questions and at least one question on media exposure, but breaking off before completing the remaining questions. Break-offs were defined as answering at least one demographic question (and were age-eligible) but none of the media questions.

**Table 1:** Call outcomes and rates for the C4H interactive voice response, random digit dial mobile phone sample [using American Association for Public Opinion Research standards [13].

**Interview (Category 1)**	
Complete (1.1)	9469
Partial (1.2)	3547
**Eligible, non-interview (Category 2)**	
Break-off/refusal (2.12)	2987
**Unknown eligibility, non-interview (Category 3)**	
No screener completed (3.21)	1196
Unknown if person is HH resident (3.3)	27626
**Not eligible (Category 4)**	
Unknown if number is valid, call did not connect (4.31)	918277
Under age (4.70)	2024
Other (Call connected but no/invalid selection) (4.9)	111402
**Total phone numbers used**	1076528
I = Complete interviews (1.1)	9469
P = Partial interviews (1.2)	3547
R = Refusal and break-off (2.1)	2987
NC = Non contact (2.2)	0
O = Other (2.0, 2.3)	0
Calculating e:^**a**^	.88
UH = Unknown household (3.1)	0
UO = Unknown other (3.2–3.9)	28822
**Response Rates**	
Response rate 1: I/(I+P) + (R+NC+O) + (UH +OU)	21.1
Response rate 2: (I+P)/(I+P) + (R+NC+O) + (UH+OH)	29.0
Response rate 3: I/((I+P) + (R+NC+O) + e(UH+UO))	22.8
Response rate 4: (I+P)/((I+P) + (R+NC+O) + e(UH+UO))	31.3
**Cooperation Rates**	
Cooperation rate 1 [&3]: I/(I+P)+R+O)	59.17
Cooperation rate 2 [&4]: (I+P)/((I+P)+R+O))	81.33
**Refusal Rates**	
Refusal rate 1: R/((I+P)+(R+NC+O) + UH + UO))	6.7
Refusal rate 2: R/((I+P)+(R+NC+O) + e(UH + UO))	7.2
Refusal rate 3: R/((I+P)+(R+NC+O))	18.7
**Contact Rates**	
Contact rate 1: (I+P)+R+O / (I+P)+R+O+NC+ (UH + UO)	35.7
Contact rate 2: (I+P)+R+O / (I+P)+R+O+NC + e(UH+UO)	38.5
Contact rate 3: (I+P)+R+O / (I+P)+R+O+NC	100.0
**Survey length, costs, and productivity**	
Average survey length (mins)	9:50
Average estimated cost per interview	USD 4.95
Telephone numbers used to get a survey start	23
Telephone numbers used to get an eligible contact	67
Telephone numbers used to get an interview *(full + partial)*	83

^**a**^e is estimated as the proportion of all respondents screened for eligibility who were eligible.

Respondents who started the survey but did not answer the age question were considered to have unknown eligibility. Ineligible respondents were under 18 years of age.

Phone numbers that were dialed but could not be confirmed as known working numbers were classified as not eligible.[14,15] These included numbers that were not in service, not working, or the survey did not play because a valid connection with the phone could not be established. Dialed numbers where the call connected at the network level but a valid connection to an individual’s mobile phone could not be confirmed also were classified as not eligible; these calls never rang on a person’s phone, went to voicemail, or were quick hang-ups. We expected a high number of not eligible calls because of the automated nature of the RDD calling system.

The productivity of the sampling approach was determined by calculating the number of phone numbers called to yield a completed interview, a survey start, and an eligible respondent. The average time for survey completion was computed using full interviews. Cost estimates were calculated for a 20-question survey in five languages, and included costs for translation, audio recording in local languages, survey build, 1-month for implementation, data management, and mobile phone airtime.

### Sample quality

To assess quality of the C4H sample, potential biases due to errors in coverage and nonresponse were examined. Data from the Ghana DHS—collected most recently in 2014 using a nationally representative sample of household face-to-face interviews with 15–49 year olds[11]—were used for comparing demographic and media exposure characteristics of the C4H sample. Questions about exposure to health messages were similar across the C4H and DHS surveys, although the C4H survey assessed exposure in the past month while the DHS survey assessed exposure over the past few months. Data from the 2014 Ghana DHS were used to benchmark ITN use among the C4H sample. The GSS census data, obtained via household sampling and data collection with people 15 and older and last undertaken in 2010 with more recent projections for 2017,[12] also was used for comparison. Differences between samples were computed as absolute values in tables; statistical testing was not performed because large sample sizes would be highly likely to yield significant findings regardless of the magnitude of differences.

## Results

### Response rates

A total of 16,003 eligible respondents participated in the survey: 9,469 completed the full interview and 3,547 completed a partial interview, while 2,987 began the survey but broke-off before answering media exposure questions (Table 1).

In order to get 46,849 people to start the survey (indicated by choosing their preferred language), 1,076,258 calls were placed; this is a productivity rate of 23 calls dialed for a survey start. Sixty-seven calls were dialed to yield an eligible contact, and 83 calls were dialed to yield an interview. The large majority of numbers dialed did not connect with an eligible respondent—many numbers were unassigned or not in service, the phone did not ring because a person was out of range to receive the call or the phone was turned off, or the call went to voicemail or was an immediate hang up. These dispositions were categorized as not eligible under Category 4 outcomes (Table 1).

AAPOR response rate 4, cooperation rate 2/4, refusal rate 2, and contact rate 2 were 31.3%, 81.3%, 7.2%, and 38.5% respectively. Notably, the large majority (81.33%) of eligible respondents completed the interview. The average survey length was 9:50 and the average estimated cost per survey was USD 4.95 (Table 1).

### Demographics

Characteristics of the C4H sample are shown in Table 2. Young men formed the majority of the sample: two-thirds of respondents were male and more than half were 18–24 years of age. The majority of respondents lived in urban areas, although there was substantial response from all ten regions in Ghana. In comparison to DHS and GSS data, C4H survey respondents were more likely to be male, younger, more urban, less likely to be married, and better educated.

**Table 2:** Demographic characteristics of national samples from 2017 C4H survey, 2014 Ghana DHS, and the 2010/2017 GSS census.

	C4H 2017 (18+ years)	Ghana DHS 2014 (15–49 years)	GSS Census 2010, 2017 (15+ years)	*Difference between C4H and DHS*	*Difference between C4H and GSS*
Sample Size	13,016	13,265	13,632,299–18,177,311		
**Sex**					
Males	66.7	29.0^b^	48.1^d^	*37*.*7*	*18*.*6*
Females	33.3	71.0	51.9		
**Age**					
15–24	55.9^a^	35.3	30.7^e^	*20*.*6*	*25*.*2*
25–34	29.6	31.0	24.4	*-1*.*4*	*5*.*2*
35–49	9.4	33.7	24.8	*-24*.*3*	*-15*.*4*
50+	5.1	—	20.2		*-15*.*1*
**Residence**					
Urban	67.7	53.5	50.9^e^	*14*.*2*	*16*.*8*
Rural	32.3	46.5	49.1		
**Region**					
Greater Accra	30.9	20.6	18.9^d^	*10*.*3*	*12*
Ashanti	23.9	18.7	19.2	*5*.*2*	*4*.*7*
Brong Ahafo	8.4	8.2	10.7	*0*.*2*	*-2*.*3*
Central	8.0	9.9	9.3	*-1*.*9*	*-1*.*3*
Northern	6.6	8.3	9.0	*-1*.*7*	*-2*.*4*
Western	6.4	11.2	8.1	*-4*.*8*	*-1*.*7*
Eastern	5.7	9.3	10.1	*-3*.*6*	*-4*.*4*
Volta	5.3	7.7	8.6	*-2*.*4*	*-3*.*3*
Upper West	2.7	2.3	2.7	*0*.*4*	*0*
Upper East	2.1	3.8	4.0	*-1*.*7*	*-1*.*9*
**Marital Status**					
Single	56.2	37.3	29.6^e^^,^^f^	*18*.*9*	*26*.*6*
Married/living with partner	39.6	54.0	57.8	*-14*.*4*	*-18*.*2*
Separated/divorced	3.0	6.7	6.5	*-3*.*7*	*-3*.*5*
Widowed	1.2	2.0	6.0	*-0*.*8*	*-4*.*8*
**Highest education**^c^					
No education	10.4	16.2	28.5^e^	*-5*.*8*	*-18*.*1*
Primary school	12.3	16.7	11.5	*-4*.*4*	*0*.*8*
Middle/JHS	24.6	41.4	36.1	*-16*.*8*	*-11*.*5*
Secondary+	52.7	25.7	23.9	*27*	*28*.*8*

^a^The C4H survey included 18+.

^b^Male respondents were interviewed in a subsample of selected households, yielding a smaller number of men than women.

^c^DHS, GSS measure highest level of education attained regardless whether that level was completed; C4H measures highest level completed.

^d^Data were computed using projected census data for 2017.

^e^Data are from 2010.

^f^Marital status was assessed for 18+.

C4H respondents reported higher radio listening and TV viewership, but less exposure to messages about family planning and malaria than DHS respondents (Table 3). Reporting of ITN use the previous night was more similar across the C4H and DHS surveys.

**Table 3:** Media use, exposure to health messages, and insecticide treated net (ITN) use in C4H and DHS samples.

Indicator/Question	C4H 2017 (18+ years)		Ghana DHS 2014 (15–49 years)		*Difference between C4H and GSS*
	N	%	N	%	
**Media use**					
Radio Listening (Listens to radio at least once a week)	13,016	79.7	13,265	59.6	*20*.*1*
TV Viewership (Watches TV at least once a week)	12,708	80.7	13,265	55.1	*25*.*6*
**Exposure to health messages**					
Family planning^a^	10,485	54.4	9,089	65.8	*-11*.*4*
Malaria prevention^b^	9,691	72.6	11,835	93.2	*-20*.*6*
**ITN use**					
Slept under an ITN last night (Children under 5)	4,577	54.3	5,801	46.6	*7*.*7*
Slept under an ITN last night (Pregnant women)	178	39.0	654	43.3	*-4*.*3*
Slept under an ITN last night (Household members)	9,527	34.0	40,337	35.7	*-1*.*7*

^a^C4H measure was: Heard or saw at least one advert or message about preventing or delaying pregnancy, including on the radio, tv, posters, billboards, or other channels in the past month. DHS measure was: Heard or saw a family planning message on radio, on television or in a newspaper or magazine in the past few months.

^b^C4H measure was: Heard or saw at least one advert or message about using insecticide treated nets to prevent malaria, including on the radio, tv, posters, billboards, or other channels in the past month. DHS measure was: Heard or saw messages that families should sleep under insecticide treated nets (ITNs) to protect them from malaria, especially pregnant women and children under age 5 in the past six months.

## Discussion

This is the first study we know of to report call outcomes from a mobile phone survey of a national sample in a developing country. The innovative interactive voice response, random digital dial, automated mobile phone data collection yielded approximately 13,000 interviews in 27 days. The average estimated cost was USD 4.95 per completed interview, which is low compared to household surveys.[16] Our response rates were comparable to other survey research methodologies, and once a person was deemed eligible for survey response, they were highly likely to complete the survey. These data add to the small but growing research base documenting that mobile phone survey research in countries like Ghana is feasible, fast, and potentially cost-effective.

As other survey research shows, RDD mobile phone surveys are an effective method for collecting health data, especially from younger populations.[17, 18] The C4H response rates are comparable to other national health surveys: the 2011 Behavioral Risk Factor Surveillance System and the 2011 National Young Adult Health Survey conducted via RDD on cell phones in the United States yielded response rates of 28% and 24%, respectively.[19] The 2012 Australian New South Wales Population Health Survey obtained a 32% response rate from mobile phone surveys,[20] and the Australian pilot RDD mobile phone survey with women yielded a 45% response rate.[18] The Ghana C4H survey is notable because of very limited reporting of population-based mobile phone survey research in LMIC.

Non-response bias refers to differences between people who did and did not respond to the survey. Nonresponse is a challenge for mobile phone surveys[17, 18, 21] and it can be difficult to disposition calls appropriately.[21] The C4H data demonstrate that the automated RDD approach in Ghana included a large number of unassigned or non-working numbers, calls that do not go through due to network congestion or poor signals, and calls that are not answered at all. Without a sampling frame listing working mobile phone numbers—which are rare, expensive, and difficult to acquire in LMIC[8], nonresponse will be sizeable and many calls will need to be placed to obtain a sample of eligible respondents. In addition, discerning noncontacts was not possible in the C4H sample because the automated call system is limited in the type of information that can be obtained from each call placed. RDD telephone surveys in higher income countries usually rely on employees who dial each phone number and can better disposition calls, but limited resources preclude this arrangement in Ghana and most LMIC. Our information about each number dialed was further limited by dialing each number only once; future automated RDD mobile phone surveys should redial non-connected numbers between 6–10 times to better ascertain final call dispositions.[21, 22] This may be especially important in LMIC where access to electricity and cost for mobile phone airtime mean that phones are frequently turned off or unreachable.

Because calls where eligibility is undetermined are common in RDD mobile phone surveys and they have a substantial impact on calculating response rates, researchers should use all available approaches to reduce the number of undetermined calls and report multiple response rates to provide a broader perspective on survey response.[19, 22] Our data demonstrate how different estimates of eligibility can lead to dramatic differences in response rates:[22, 23] the AAPOR automated calculation of ‘e’ that is an estimate of how many calls of unknown eligibility are eligible was tiny at .015 and produced response metrics that appeared too high. Therefore, we chose a conservative approach to estimating ‘e’ and computed it as the proportion of all respondents screened for eligibility who were known to be eligible at .88. Although this adjustment reduced our response rates, it was more sensible and produced rates that have greater face validity.

Once phones are answered, language, literacy, cost, time, or availability concerns may lead to refusals. The C4H interview required respondents to select the survey language prior to the introduction greeting stating the purpose of the call, which likely lowered call continuation. The C4H survey had a low overall refusal rate, although comparisons with the Ghana DHS highlight the propensity of mobile phone surveys to oversample male, urban, younger, and better educated respondents at the expense of female, rural, older, and less educated populations. This demonstrated bias in coverage—referring to how well a sample matches the larger population—concurs with data showing that young people, men, and urban residents are more likely to own mobile phones.[6, 24] In particular, women are substantially less likely than men to own a mobile phone globally and in Africa,[25] and women may find that responding to mobile phone surveys is especially inconvenient given demands associated with caring for children and household management. Research is needed to test methods for better engaging women and rural residents in mobile phone surveys, such as using incentives, calling at certain times, survey pre-notification,[26, 27] or more motivational call greetings.

When mobile phone penetration is high, coverage bias is reduced: experimental data from four African countries suggest that as mobile phone ownership increases, data obtained through mobile phone surveys increasingly approximates data obtained via household surveys.[26] Ghana in particular provides a promising environment for mobile phone survey research. It was an early adopter of mobile phones and services, has one of the most vibrant mobile phone markets in Africa, and data from 2014 showed that 83% of adults owned a mobile phone[24] and in 2015 there were 130 mobile phone subscriptions per 100 people.[28] The RDD mobile phone survey methodology may be less suitable for countries with lower rates of mobile phone access and use. Additional research is needed to assess whether the results from Ghana generalize to other developing countries.

The data showed that C4H respondents were more likely to listen to the radio and view television than DHS respondents. This result is reasonable given that urban, male, younger, and more educated Ghanaians use media more often than others[12] and there was a higher proportion of these groups in the C4H sample. On the other hand, exposure to family planning and malaria messages was lower in the C4H survey than the DHS; these results are reasonable given that the C4H questions assessed health message exposure in the last month while the DHS questions referred to the past six months. C4H results also might indicate that young people are less targeted or concerned about health issues, as DHS data show that younger people report less exposure to health messages than older respondents.[11]

We chose not to weight the data, as reporting our methods without weighting is appropriate given the innovative nature of the research design and the need to share these results with the global research community.[21] However, adjusting data from mobile phone samples, and in particular by sex and age, has proven to reduce noncoverage bias by yielding demographic characteristics[26] and health indicators that are quite comparable to household samples.[10, 29] Future research using mobile phone surveys in in Ghana or other LMIC should consider weighting raw data to more accurately estimate health indicators for nationally representative samples.[7]

## Conclusions

Worldwide, utilization of mobile phones for data collection and research is increasing. Although RDD mobile phone surveys are unlikely to fully replace door-to-door demographic and health surveys, monitoring and evaluation staff should consider the utility of RDD as an alternative or supplemental data collection method. This survey methodology is especially suitable for reaching populations with high access to mobile phones such as in Ghana, and people 35 and younger, from urban or peri-urban areas, and males. Gauging the feasibility of RDD in LMIC will be facilitated by standardized reporting of response rates and call outcomes using widely accepted standards such as AAPOR. Disclosure of survey implementation costs using comparable costing elements, for both mobile phone and face to face surveys, also is needed. Lower-middle income countries such as Ghana, which face a gap in donor assistance for population-based surveillance and national censuses[30] and have relatively broad access to mobile phones,[26] may present a prime opportunity for RDD mobile phone surveys.

## Supporting information

S1 Dataset Dataset for full and partial interviews. (XLSX)
(XLSX)

S1 Appendix Survey script in English. (DOCX)
(DOCX)

## Abbreviations

AAPOR: American Association of Public Opinion Research
C4H: Communicate for Health project
DHS: Demographic and health survey
GSS: Ghana Statistical Service
GHS: Ghana Health Service
ITN: Insecticide-treated bed net
LMIC: Low and middle income countries
MNO: Mobile network operators
IVR: Interactive voice response
RDD: Random digit dialing
