# Supplementary material for: Survey research with a random digit dial national mobile phone sample in Ghana: Methods and sample quality
Source: PLoS One. 2018 Jan 19;13(1):e0190902. doi: 10.1371/journal.pone.0190902 (PMC5774708; doi:10.1371/journal.pone.0190902)
Supplement: S1 Appendix — (DOCX) (DOCX) [file pone.0190902.s002.docx]

LANGUAGE SELECTOR

*Hello, I am calling from Ghana Health Service. I also speak Ga, Ewe, Dagbani, and English.*

*To continue in English, press 1. To continue in Ga, press 2. To continue in Twi, press 3. To continue in Ewe, press 4. To continue in Dagbani press 5*

*INTRO*

*Hello, we are conducting research for the Ghana Health Service to learn more about Ghanaians health practices. This call is free and your responses are confidential. We will never ask for your name. I would like to ask you a few questions about your health. The questions I will ask are pre-recorded and you will answer by pressing the numbers on your phone. Please listen to all the answer options; you can press [*] to repeat the question at any time. You must be 18 or older to participate****.***

*Press 1 to give your input now.*

*Press 2 if you cannot talk now. You are welcome to call me back at this number at any time in the next few days to give your input.*

Demographics

| Q# | Question | Responses | Directions |
| --- | --- | --- | --- |
| D.1 | How old are you? | 1. <18 2. 18-24 3. 25-35 4. 36 to 49 5. 50 and older | 🡪Ineligible: Closing message |
| D.2 | Are you female or male? | 1. Female 2. Male |  |
| D.3 | In which region do you primarily reside? | 1. Ashanti 2. Greater Accra 3. Eastern 4. Western 5. Brong Ahafo 6. Northern 7. Central 8. Volta 9. Upper East 10. Upper West |  |
| D.4 | How old is your youngest child? | 1. I don’t have any children 2. Under 5 years 3. Between 5 and 17 4. 18 or older |  |
| D.5a | [Women only] Are you currently pregnant? | 1. Yes 2. No | 🡪D.6a  🡪D.7 |
| D.5b | [Men only] Do you have a female partner who is currently pregnant? | 1. Yes 2. No | 🡪D.6b  🡪D.7 |
| D.6a | [Women only] How many months pregnant are you? | 1. I’m not currently pregnant 2. 1-3 months 3. 4-6 months 4. 7-9 months 5. I don’t know how far along I am |  |
| D.6b | [Men only] How many months pregnant is your partner? | 1. Partner is not pregnant 2. 1-3 months 3. 4-6 months 4. 7-9 months 5. I don’t know how far along she is |  |
| D.7 | What is the highest level of education you have completed? | 1. No education 2. Primary 3. Middle/JSS/JHS 4. Secondary/SSS/SHS/ Vocational/Technical 5. Tertiary or higher |  |
| D.8 | Are you currently single, married or living with a partner, separated or divorced, or widowed? | 1. Single 2. Married or living with a partner 3. Separated or divorced 4. Widowed |  |
| D.9 | Do you live in an urban or rural community? | 1. Urban 2. Rural |  |

Exposure to Health Messages

| Q# | Question | Responses | Directions |
| --- | --- | --- | --- |
| E.1 | In the last 7 days, how often did you listen to the radio? | 1. Every day 2. Most days 3. A few days 4. Not at all |  |
| E.2 | In the last 7 days, how often did you watch television? | 1. Every day 2. Most days 3. A few days 4. Not at all |  |
| E.3 | Have you heard of the GoodLife Live it Well campaign? The campaign has messages like this: (include an audio clip with new branding) | 1. Yes 2. No 3. Not sure |  |
| SO4 | In the past month, about how many messages or adverts have you seen or heard about handwashing, including on the radio, tv, posters, billboards, or other channels? | 1. More than 10 messages or adverts 2. 6-10 messages or adverts 3. 1-5 messages or adverts 4. Zero messages or adverts |  |
| SO3 | In the past month, about how many messages or adverts have you seen or heard about preventing or delaying pregnancy, including on the radio, tv, posters, billboards, or other channels? | 1. More than 10 messages or adverts 2. 6-10 messages or adverts 3. 1-5 messages or adverts 4. Zero messages or adverts |  |
| SO5/6 | In the past month, about how many messages or adverts have you seen or heard about using insecticide treated nets to prevent malaria, including on the radio, tv, posters, billboards, or other channels? | 1. More than 10 messages or adverts 2. 6-10 messages or adverts 3. 1-5 messages or adverts 4. Zero messages or adverts |  |

Insecticide-Treated Bednet Use

| SO6 | Last night, did you sleep under an insecticide treated net? | 1. Yes 2. No |  |
| --- | --- | --- | --- |
|  | Parents of children under five only |  |  |
| SO5.1 | How many children under five live in your household? | 1. 1 2. 2. 3. 3 4. 4 5. 5 or more 6. None |  |
| SO5.2 | Last night, how many of the children under 5 in your household slept under an insecticide treated net? | 1. 1 2. 2 3. 3 4. 4 5. 5 or more 6. None |  |
